# NeuroDetect: Deep Learning-Based Signal Detection in Phase-Modulated Systems with Low-Resolution Quantization

**DOI:** 10.3390/s25103192

**Published:** 2025-05-19

**Authors:** Chanula Luckshan, Samiru Gayan, Hazer Inaltekin, Ruhui Zhang, David Akman

**Affiliations:** 1 Department of Electronic and Telecommunication Engineering, University of Moratuwa, Moratuwa 10400, Sri Lanka; luckshangwcm.20@uom.lk (C.L.); samirug@uom.lk (S.G.); 2 School of Engineering, Macquarie University, North Ryde, NSW 2109, Australia; 3 Institute of Advanced Study in Mathematics, Harbin Institute of Technology, Harbin 150001, China; ruhui_zhang@hit.edu.cn; 4 Lifelong Learning, University of New South Wales, Kensington, NSW 2052, Australia; d.akman@unsw.edu.au

**Keywords:** low-resolution ADCs, machine learning, deep learning, model-free, signal detection, maximum likelihood detection, energy efficiency

## Abstract

This manuscript introduces NeuroDetect, a model-free deep learning-based signal detection framework tailored for phase-modulated wireless systems with low-resolution analog-to-digital converters (ADCs). The proposed framework eliminates the need for explicit channel state information, which is typically difficult to acquire under coarse quantization. NeuroDetect utilizes a neural network architecture to learn the nonlinear relationship between quantized received signals and transmitted symbols directly from data. It achieves near-optimum performance, within a worst-case 12% margin of the maximum likelihood detector that assumes perfect channel knowledge. We rigorously investigate the interplay between ADC resolution and detection accuracy, introducing novel penalty metrics that quantify the effects of both quantization and learning errors. Our results shed light on the design trade-offs between ADC resolution and detection accuracy, providing future directions for developing energy-efficient high-speed and wideband wireless systems.

## 1. Introduction

### 1.1. Background and Motivation

Next-generation wireless systems, including ultra-massive multiple-input multiple-output (MIMO) arrays, millimeter-wave (mmWave) networks, and emerging sub-THz technologies, are pushing the limits of data rate, bandwidth, reliability, and latency requirements [1,2,3]. As these systems grow in scale and complexity, receiver power consumption emerges as a critical design bottleneck. Among the receiver components, ADCs contribute a significant fraction of the total power budget, particularly in high-speed and wideband wireless systems [4,5].

The power consumed by ADCs scales exponentially with resolution and linearly with sampling rate [6]. As a result, employing high-resolution ADCs in systems with high sampling rates can lead to substantial energy overhead. For instance, a high-speed ADC operating at or above 20 GSamples/s with 8–12 bits of resolution typically consumes around 500 mW. Since each RF chain requires two ADCs, this results in approximately 1 W per RF chain. In a future mmWave massive MIMO system with 256 RF chains (512 ADCs in total), the aggregate power consumption can exceed 256 W, posing a major challenge for practical deployment [7]. To address this issue, low-resolution ADCs have emerged as a promising solution, significantly reducing power consumption by limiting the number of quantization bits [8,9].

While low-resolution ADCs offer substantial energy savings, they also introduce significant quantization-induced nonlinearities that fundamentally alter the input–output relationship of the communication system [10]. These nonlinear distortions undermine the assumptions underpinning traditional coherent detection methods, which typically depend on accurate channel state information (CSI). Estimating CSI becomes considerably more difficult under coarse quantization, due to the limited information content in the quantized observations [11]. Consequently, any mismatch or inaccuracy in channel estimation can lead to severe performance degradation, particularly in systems employing higher-order modulation schemes or operating in fast-fading environments.

A range of prior studies, such as [12], have explored *model-based* detection strategies, including maximum likelihood (ML) detection and sphere decoding, to mitigate the effects of quantization noise. While these methods are theoretically near-optimum, they often rely on the assumption of perfect or near-perfect CSI and become computationally infeasible as system dimensions and modulation orders increase. To address the trade-off between detection performance and practical complexity, deep learning (DL)-based detection techniques have emerged as compelling alternatives, offering great potential as data-driven, low-complexity solutions without explicit reliance on channel models.

The DL-based techniques enable the development of parametric models that learn system dynamics and signal detection rules directly from data. This class of methods, known as *model-driven* approaches, incorporates a priori domain knowledge into the network architecture while learning model parameters from training data [13]. By leveraging model structure, these approaches can approximate ML detection, achieving reduced computational complexity and improved numerical stability, especially in high signal-to-noise ratio (SNR) regimes [14].

Despite their advantages, model-driven approaches often depend on partial or idealized model knowledge, limiting their ability to generalize when real-world conditions diverge from training assumptions. In practical wireless environments, where channel characteristics may be highly dynamic, nonlinear, or poorly understood, these methods may fail to deliver robust performance. This limitation motivates the need for a *model-free* approach to signal detection, in which the underlying channel input–output relationship is learned directly from data, without relying on prior model assumptions.

To address these challenges, we propose NeuroDetect, a model-free DL-based signal detection framework designed for receivers equipped with low-resolution ADCs. NeuroDetect operates entirely without explicit channel knowledge and learns to map quantized observations to transmitted symbols directly from data. Our approach capitalizes on the representational power of deep neural networks to capture the underlying nonlinear relationship introduced by low-resolution quantization. By training on a small number of labeled examples within each channel coherence interval, NeuroDetect effectively eliminates the need for channel estimation, thereby offering robust performance across a range of fading environments.

### 1.2. Main Contributions

The key contributions of this work are summarized as follows:

- **Model-Free Detection:** We introduce a deep neural network architecture that learns to detect symbols under phase quantization without requiring explicit CSI. The proposed solution complements existing model-based signal detection approaches by offering a robust alternative for scenarios where CSI is unavailable at the receiver with low-resolution quantization.
- **Near-Optimum Performance:** We demonstrate that NeuroDetect achieves symbol error rates within 12% of the ML detector, which assumes perfect CSI. This is the worst-case performance gap, and we show that the gap is much smaller in almost all scenarios we studied. This result is significant as it shows that near-optimum detection is attainable without the overhead of channel estimation. A key merit of NeuroDetect is to show that a lightweight and fully-connected architecture, if trained model-free, can close the gap to optimal ML detection under severe quantization errors, without the need for custom layers or specialized activation functions.
- **Asymptotic Optimality:** We show that the proposed DL-based detector achieves the optimum asymptotic error decay rate for different quantization levels, revealing a characteristic ternary behavior in diversity order.
- **Penalty Metrics:** We develop new metrics to quantify the learning and quantization penalties, offering insights into how the number of bits affects detection accuracy and power requirements.

### 1.3. Related Work

This section provides an overview of prior signal detection strategies for low-resolution ADC-based receivers. We first discuss a range of classical approaches and their inherent limitations. We then review DL-based solutions.

#### 1.3.1. Classical Approaches

Over the past few years, numerous classical schemes have been developed for signal detection in low-resolution ADC-based wireless systems. Early work in [15] introduced a linear minimum-mean-square-error (LMMSE) receiver, which outperforms the conventional Wiener filter. However, this approach has the drawback of having error floors in the high SNR regime.

To overcome this limitation, subsequent research proposed the Bussgang LMMSE (BLMMSE) estimator, extending classical linear data detectors such as maximum ratio combining (MRC) and zero-forcing (ZF) via Bussgang decomposition [16]. This method was later adapted to the multi-bit scenario in [17], demonstrating improved performance over LMMSE. Nonetheless, error floors persist in the high SNR regime.

Assuming perfect CSI at the receiver, ML data detection has been investigated for one-bit quantized MIMO systems in [18,19] and extended to one-bit MIMO-OFDM systems in [20]. However, the computational complexity of the method in [18] increases exponentially with the signal constellation size, number of transmit antennas, and overall network dimensions, making it impractical for real-world implementations. To mitigate this issue, ref. [21] introduced a near-optimal ML detector based on convex optimization techniques. Although this method outperforms ZF, MRC, and LMMSE detectors in terms of SEP, simulation results indicate that it still suffers from an error floor at high SNR.

In [22], a sphere decoding-based near-ML detection method, termed one-bit sphere decoding (OSD), was presented for massive MIMO systems with one-bit ADCs. OSD reduces complexity by decomposing the received signal vector into multiple sub-vectors, thus balancing performance and computational demands. However, the complexity still grows exponentially with the number of transmit antennas and the constellation size.

Subsequent work in [23] addressed this scalability challenge by developing signal detection algorithms for one-bit MIMO systems under both perfect and statistical CSI, achieving near-optimum performance compared to the oracle lower bound in [24]. This method uses the Laplace approach to estimate the channel model with a Gaussian distribution. However, it does not work well for all channel models, and the accuracy drops in real-world situations with more complex models.

Several studies, including our previous work, have investigated low-resolution phase quantization for *M*-PSK modulation schemes [11,12,25,26,27]. These works propose various detection techniques and demonstrate their effectiveness through Monte Carlo simulations. Notably, the simulations in [26] reveal that, under QPSK modulation with 8-bit phase quantization, a non-coherent block-fading channel can retain approximately 80–85% of the mutual information attainable with fully unquantized observations, incurring only a modest 2–3 dB increase in SNR. Further, ref. [27] shows that PSK achieves capacity under various channel models when phase quantization is employed at the receiver output.

#### 1.3.2. DL-Based Approaches

DL-based methods offer a compelling alternative for the classical approaches, delivering superior performance in practical scenarios while reducing computational complexity [28]. These methods have already demonstrated remarkable capabilities in channel estimation, equalization, and signal detection, making them a viable solution for future wireless systems [29,30,31,32].

A supervised learning approach for blind signal detection was proposed in [33] to learn the empirical probability density function for each symbol with low-resolution quantization. Here, three blind detection methods were introduced: Empirical Maximum Likelihood Detection (e-MLD), Minimum Mean-Distance (MMD), and Minimum Center Detection (MCD). These methods are related to *K*-nearest neighbors classification and nearest centroid classification. It has been shown that MCD performs better than MMD and e-MLD. However, like classical schemes, the practical implementation is limited due to high complexity.

The same authors proposed a more efficient supervised learning-aided successive interference cancellation method to reduce both training overhead and complexity [34]. However, the efficiency of this method depends on the training sequence, which requires a long sequence of data to effectively learn the function approximator. To address this performance degradation, a supervised and semi-supervised framework has been introduced in [35], which uses a cyclic redundancy check for error control.

Several other model-driven DL approaches were proposed for signal detection and channel estimation with low-resolution ADCs. The Few-Bit Massive MIMO Channel Estimation Network (FBMCENet) and the Few-Bit Massive MIMO Data Detection Network (FBM-DetNet) [36] outperform the detector in [35] with QPSK and 16-QAM constellations with an SNR of 10 dB when perfect CSI is available. However, their complexity remains a major drawback. To address this, the same authors proposed the One-Bit Massive MIMO Data Detection Network (OBMNet), which integrates DL with conventional signal processing techniques [37]. By using a sigmoid approximation of the Gaussian cumulative distribution function, OBMNet achieves lower complexity compared to other proposed methods.

These model-driven approaches require the availability of CSI as well as prior knowledge of the system parameters. Hence, using model-driven approaches in a dynamically changing wireless communication system poses a challenge. By bridging the gap between model-free and model-driven approaches, the authors in [38] proposed LoRD-Net (Low Resolution Detection Network), a hybrid system that learns to carry out blind symbol detection from one-bit measurements.

Beyond signal detection, DL was also applied to pilot design and channel estimation in mixed-ADC massive MIMO systems [39,40]. These papers reduce pilot overhead and further highlight the potential of DL-based techniques in wireless communications with low-resolution ADCs. Bidirectional LSTM (BiLSTM) models demonstrated significant improvements in channel estimation for MIMO-OFDM low-resolution ADC systems by leveraging bi-directional training [41,42].

## 2. System Setup

### 2.1. Channel Model and Signal Modulation

We focus on the point-to-point wireless channel with flat-fading. The received discrete-time baseband equivalent signal *Y* is given by

(1) $$\begin{array}{c} Y=\sqrt{\mathrm{SNR}}\cdot HX+W, \end{array}$$

where X∈C⊂C is the transmitted signal, C is the constellation set of information signals in C (complex plane), SNR is the ratio of the transmitted signal energy to the additive white Gaussian noise (AWGN) spectral density, H∈C is the unit power channel gain between the transmitter and the receiver, and *W* is the circularly-symmetric zero-mean unit-variance AWGN. In (1), the prefactor $\sqrt{\mathrm{SNR}}$ follows a standard normalization convention that keeps *X* unit-energy and *W* unit-variance for analytical tractability and simplicity of the exposition. We will assume that $C=\{e^{j\pi \left(\frac{2\gamma +1}{M}-1\right)}{\}}_{\gamma =0}^{M-1}$ throughout the paper. This represents the conventional *M*-ary phase shift keying (*M*-PSK) constellation. For simplicity, we restrict our analysis to cases where *M* is an integer power of 2. This setup is also adopted in our previous work [12], and we use those analytical results as a benchmark for our study.

### 2.2. Receiver Architecture

**Figure 1:**
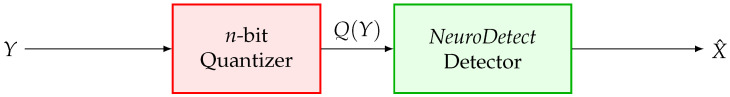
General receiver architecture with the low-resolution quantization. Here, NeuroDetect is the proposed DL-based framework for signal detection.

As illustrated in Figure 1, the receiver architecture is based on a low-resolution ADC.

In this setup, the received signal *Y* is first sent through a low-resolution quantizer, and the resulting quantized received signal Q(Y) is then used to estimate the transmitted signal *X*.

In this work, we use a phase quantizer. The adoption of phase quantization in our receiver architecture is driven by two key factors. First, since channel impairments typically manifest as phase rotations in transmitted signals, the quantization and decision regions for *M*-PSK modulation can be naturally represented as convex cones in the complex plane [12], eliminating the need for automatic gain control. Second, phase quantizers can be efficiently implemented using one-bit ADCs composed of simple comparators, which operate with minimal power consumption, typically in the milliwatt range.

If *n* bits are used to quantize *Y*, the quantizer Q(·) divides the complex domain C into $2^{n}$ quantization regions and outputs the index of the region in which *Y* lies as an input to the detector. Formally, we declare Q(Y)=q if $Y\in R_{q}$ for $q\in [0:2^{n}-1]$, where $R_{q}\subseteq C$ is the *q*th quantization region. Since information is encoded in the phase of *X* with the above choice of constellation points, $R_{q}$ is chosen to be the convex cone given by

(2) $$\begin{array}{c} R_{q}=\left\{z\in C:\frac{2\pi }{2^{n}}q\leq \mathrm{Arg}\left(z\right)+\pi \leq \frac{2\pi }{2^{n}}\left(q+1\right)\right\}. \end{array}$$

The receiver architecture outlined so far is similar to the one presented in [12]. The key distinction of our work lies in the implementation of a DL signal detector. Our DL architecture utilizes the quantized signal information Q(Y) to predict the transmitted signal *X*, without having any channel knowledge. In the following section, we detail the design and functionality of the proposed DL-based signal detector.

## 3. Deep Learning-Based Signal Detection

This section presents our proposed DL-based signal detector designed to achieve near-optimum performance in low-resolution quantization scenarios, without relying on explicit channel knowledge. The ML detector proposed in [12] relies on channel information to generate ML estimates of the transmitted data symbols. On the other hand, our DL-based detector is a fully data-driven solution, utilizing a deep neural network to extract meaningful features from quantized signal inputs. We provide the details for the model-free DL framework for signal detection with low-resolution quantization, our DL-based signal detector, its neural network architecture, and the corresponding training methodology in the following subsections.

### 3.1. Deep Learning for Signal Detection with Low-Resolution Quantization

**Figure 2:**
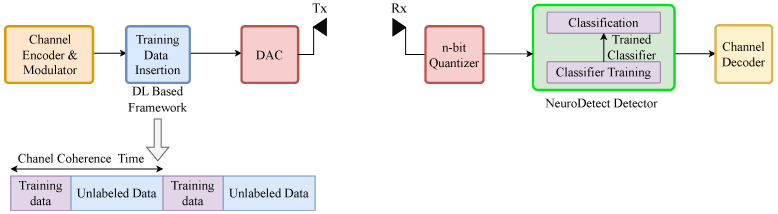
Model-free deep learning framework for signal detection with low-resolution quantization. Here, DAC is the digital-to-analog converter.

We adopt the model-free signal detection framework illustrated in Figure 2, originally proposed in [13], for symbol detection in low-resolution wireless communication systems. The input–output relationship is highly nonlinear with low-resolution quantization, which is a challenge for signal detection using traditional detection methods. Accurate channel estimation is also another important challenge, due to the low number of quantization bits used at the receiver to estimate the channel. The model-free signal detection framework overcomes these challenges by learning a near-optimum signal detector directly from training data, and, hence, eliminating the need for channel estimation while capturing input–output non-linearity.

Within this framework, we divide the transmitted symbols during a channel coherence interval into two categories: *training data* and *unlabeled data*. The training data, which are known to both the transmitter and receiver, serve as labeled data for training a classifier to function as a signal detector at the receiver. We call the phase during which we train the classifier at the receiver the *Training Phase* (TP). We use the quantized received signals $Q\left(Y\right)$ as input features and the transmitted symbols *X* as target values during the TP to train the classifier.

The unlabeled data consist of transmitted symbols that are unknown to the receiver. To detect these symbols, the receiver uses the trained classifier with optimized parameters. We refer to this stage as the Data Transmission Phase (DP). During the DP, our signal detector processes the quantized received signal $Q\left(Y\right)$, assigns a likelihood to each possible transmitted symbol based on the observation $Q\left(Y\right)$, and selects the most probable symbol as the estimate $\hat{X}$ of the transmitted symbol *X*. Unlike traditional methods or model-driven AI approaches [38,43], this framework operates without explicit channel state information.

We employ a DL-based classifier for signal detection at the receiver side of this framework. Our DL-based classifier consists of an input layer that gets $Q\left(Y\right)$ as an input, four hidden layers, and a SoftMax output layer (outputting the likelihoods for the transmitted data symbols). The architecture and implementation details of our proposed DL signal detector are given in Section 3.3.

### 3.2. Deep Learning-Based Signal Detector

**Figure 3:**
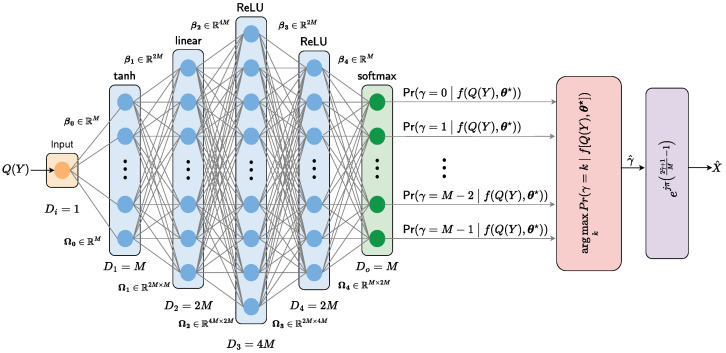
The proposed DL-based signal detector architecture for receivers with low-resolution quantization.

Figure 3 shows the architecture of our DL-based signal detector. The proposed DNN classifies the received quantized signal Q(Y) into one of the *M* possible transmitted signals, given by

$$\begin{array}{c} X_{\gamma }=e^{j\pi \left(\frac{2\gamma +1}{M}-1\right)}\;\mathrm{for}\;\gamma =0,1,\ldots ,M-1. \end{array}$$

As the transmitted symbol depends only on γ, the proposed architecture is designed to predict γ by observing the quantized received signals.

To build our DL-based detector, we define a model $f\left(Q(Y),\theta \right)$ with parameters θ, which processes an input Q(Y) from a training dataset of *I* feature/label pairs ${\left\{\left(Q{\left(Y\right)}^{\left[i\right]},\;\gamma^{\left[i\right]}\right)\right\}}_{i=1}^{I}$. This model produces a conditional probability distribution $\mathrm{Pr}\left(\gamma |\lambda \right)$, where λ are distribution parameters.

Because γ can take on any of the *M* discrete values $\left\{0,1,\ldots ,M-1\right\}$, we adopt a categorical distribution governed by $\lambda ={\left[\lambda_{0},\lambda_{1},\ldots ,\lambda_{M-1}\right]}^{⊤}$. Hence,

$$\mathrm{Pr}\left(\gamma =k|\lambda \right)\;=\;\lambda_{k},\;k=0,1,\ldots ,M-1.$$

Our model learns to predict λ given Q(Y) and, thus,

$$\mathrm{Pr}\left(\gamma |\lambda \right)\;=\;\mathrm{Pr}\left(\gamma \;|\;f\left(Q(Y),\theta \right)\right).$$

The parameters $\lambda_{1},\ldots ,\lambda_{M-1}$ are restricted to lie between zero and one and must sum to one to form a valid probability distribution. Therefore, we pass the *M* outputs of the network through a softmax function that ensures these constraints are satisfied. Then, we have

(3) $$\begin{array}{c} \mathrm{Pr}\left(\gamma =k|f\left(Q(Y),\theta \right)\right)={\mathrm{softmax}}_{k}\left(f\left(Q(Y),\theta \right)\right), \end{array}$$

where ${\mathrm{softmax}}_{k}\left(z\right)=\frac{e^{z_{k}}}{\sum_{m=0}^{M-1}e^{z_{m}}}$.

To train the model, we find the network parameters θ that minimize the negative log-likelihood loss function $L\left(\theta \right)$ over the training dataset pairs ${\left\{\left(Q{\left(Y\right)}^{\left[i\right]},\gamma^{\left[i\right]}\right)\right\}}_{i=1}^{I}$:

(4) $$\begin{array}{cc} L\left(\theta \right) & =-\sum_{i=1}^{I}\log\left({\mathrm{softmax}}_{\gamma^{\left[i\right]}}\left(f\left(Q{\left(Y\right)}^{\left[i\right]},\theta \right)\right)\right),\\ \; & =-\sum_{i=1}^{I}\left(f_{\gamma^{\left[i\right]}}\left(Q{\left(Y\right)}^{\left[i\right]},\theta \right)-\log\left(\sum_{m=0}^{M-1}\exp\left(f_{m}\left(Q{\left(Y\right)}^{\left[i\right]},\theta \right)\right)\right)\right), \end{array}$$

where $f_{m}\left(Q(Y),\theta \right)$ denotes the output of the *m*-th branch of the softmax layer in Figure 3. This loss function is also known as the multiclass cross-entropy loss.

For a point estimate of γ, we take the most probable category

(5) $$\begin{array}{cc} \hat{\gamma } & =\underset{k}{\arg\max}\;\mathrm{Pr}\left(\gamma =k|f\left(Q\left(Y\right),\theta^{★}\right)\right), \end{array}$$

where $\theta^{★}=\underset{\theta }{\arg\min}\;L\left(\theta \right)$. Then, the estimated transmitted signal is given by

(6) $$\begin{array}{c} \hat{X}=\exp\left(j\pi \left(\frac{2\hat{\gamma }+1}{M}-1\right)\right). \end{array}$$

### 3.3. NeuroDetect Architecture

The details of our DL-based signal detector, which we name *NeuroDetect*, are given below. The NeuroDetect architecture comprises a fully connected network with four hidden layers, as illustrated in Figure 3. The key components are as follows:

- **Input Layer:** Receives the quantized received signal Q(Y), which is the output of the low-resolution ADC.
- **Hidden Layers:** Four fully connected layers, configured as follows:
  - Layer 1: Consists of neurons with a tanh activation function.
  - Layer 2: Consists of neurons with a linear activation function.
  - Layers 3 and 4: Consist of neurons with ReLU activation function.
- **Output Layer:** Consists of neurons with a softmax function that outputs
  $$\mathrm{Pr}\left(\gamma =k|f\left(Q(Y),\theta \right)\right),$$
  where k=0,1…M−1.
- **Inference:** To estimate the transmitted symbol $\hat{X}$, we first take the most probable category $\hat{\gamma }=\underset{k}{\arg\max}\;\mathrm{Pr}\left(\gamma =k|f\left(Q\left(Y\right),\theta^{★}\right)\right)$, using the optimum parameters $\theta^{★}$. Then, we set the predicted symbol $\hat{X}$ to $\hat{X}=\exp\left(j\pi \left(\frac{2\hat{\gamma }+1}{M}-1\right)\right)$.

The main logic that underpins our proposed architecture is to have a design that is capable of handling the challenges posed by low-resolution quantization, ensuring robust performance without access to channel state information. In particular, unlike conventional detectors that depend on explicit channel knowledge, NeuroDetect is *model-free*, utilizing quantized received signals Q(Y) to predict the transmitted signals $\hat{X}$.

A key feature of our DL detector is that it operates without access to channel information in either the training phase (TP) or the data detection phase (DP). Our method relies on the assumption that the wireless channel varies slowly relative to the signal transmission rate, which is common in practical scenarios where coherence times are long compared to symbol durations. This slow variation allows the channel to be treated as approximately constant throughout both the training and detection phases of signal recovery, even though it remains random and unknown to the receiver.

#### 3.3.1. Hidden Layers of NeuroDetect

We denote the input dimension as $D_{i}=1$ and the output dimension as $D_{o}=M$. The dimensions of the hidden layers are $D_{1}=M$, $D_{2}=M$, $D_{3}=4M$, and $D_{4}=2M$. We define the vector of hidden units at layer *l* as $h_{l}$, the vector of biases that contribute to the hidden layer l+1 as $\beta_{l}\in R^{D_{l+1}}$, and the weights that are applied to the *l*-th layer and contribute to the (l+1)-th layer as $\Omega_{l}\in R^{D_{l+1}\times D_{l}}$, where $D_{l}$ is the dimension of the corresponding *l*th layer. Then, the NeuroDetect network $f\left(Q(Y),\theta \right)$ can be written as

$$\begin{array}{cc} h_{1} & =a_{1}\left(\beta_{0}+\Omega_{0}\;Q\left(Y\right)\right),\\ h_{2} & =a_{2}\left(\beta_{1}+\Omega_{1}\;h_{1}\right),\\ h_{3} & =a_{3}\left(\beta_{2}+\Omega_{2}\;h_{2}\right),\\ h_{4} & =a_{4}\left(\beta_{3}+\Omega_{3}\;h_{3}\right)\\ f\left(Q(Y),\theta \right) & =\beta_{4}+\Omega_{4}\;h_{4}, \end{array}$$

where $a_{1}\left(z\right)=\tanh\left(z\right)$, $a_{2}\left(z\right)=z$, $a_{3}\left(z\right)=\mathrm{ReLU}\left(z\right)$, and $a_{4}\left(z\right)=\mathrm{ReLU}\left(z\right)$. Here, to enable the learning of complex, nonlinear relationships between the inputs and output, we use the rectified linear unit $\mathrm{ReLU}\left(z\right)={\left(\max\left(0,z_{i}\right)\right)}_{i=1}^{\dim\left(z\right)}$, and $\tan h\left(z\right)={\left(\frac{e^{z_{i}}-e^{-z_{i}}}{e^{z_{i}}+e^{-z_{i}}}\right)}_{i=1}^{\dim\left(z\right)}$ and $\dim\left(z\right)$ denote the dimension of the input vector **z**. The parameters θ of the model comprise all the weight matrices and bias vectors $\theta ={\left\{\beta_{l},\Omega_{l}\right\}}_{l=0}^{4}$.

#### 3.3.2. Training Dataset Generation

We generate *I* training samples ${\left\{\left(Q{\left(Y\right)}^{\left[i\right]},\gamma^{\left[i\right]}\right)\right\}}_{i=1}^{I}$ synthetically, by generating $\gamma^{\left[i\right]}$ as a uniform random integer between 0 and M−1, substituting $X=\exp\left(j\pi \left(\frac{2\gamma^{\left[i\right]}+1}{M}-1\right)\right)$, and generating $Q{\left(Y\right)}^{\left[i\right]}$ by using the channel model (1) and quantizing the received signals according to (2). The channel is assumed to be fixed (but unknown) for the training samples.

#### 3.3.3. One-Hot Encoding of Labels

While training NeuroDetect, we use one-hot encoding to generate the output label of the *i*-th training sample $1_{\gamma^{\left[i\right]}}\in R^{M}$. Then the *m*-th element of $1_{\gamma^{\left[i\right]}}$ is given by

(7) $$\begin{array}{cc} 1_{\gamma^{\left[i\right]}}\left(m\right) & =\left\{\begin{array}{cc} 1 & \mathrm{if}\;m=\gamma^{\left[i\right]}+1,\\ 0 & \mathrm{otherwise}, \end{array}\right. \end{array}$$

where i=1,…,I and m=1,…,M.

#### 3.3.4. Parameter Optimization

The parameter optimization in our model-free detection framework is performed using the Adaptive Moment Estimation (ADAM) algorithm. We adopt ADAM [44] because it offers several advantages that align well with the characteristics of our problem. Specifically, ADAM adaptively adjusts the learning rate for each parameter using first- and second-order moment estimates, making it particularly effective in training scenarios involving noisy gradients, such as those arising from limited training data within each channel coherence block. Moreover, the underlying objective function in our framework is highly non-convex, and ADAM’s stable convergence behavior in such settings is well established both theoretically and empirically [45,46]. These properties make ADAM especially suitable for our model, which must train efficiently from small, rapidly changing data in fading channels.

The iterative process continues until convergence, ensuring optimal weights for all layers. The ADAM algorithm adds momentum to both the estimate of the gradient and the squared gradient according to

(8) $$\begin{array}{c} m_{t+1}\leftarrow \alpha_{1}\cdot m_{t}+\left(1-\alpha_{1}\right)\frac{\partial L\left(\theta_{t}\right)}{\partial \theta }, \end{array}$$

(9) $$\begin{array}{c} v_{t+1}\leftarrow \alpha_{2}\cdot v_{t}+\left(1-\alpha_{2}\right){\left(\frac{\partial L\left(\theta_{t}\right)}{\partial \theta }\right)}^{2}, \end{array}$$

where $\alpha_{1}\in \left[0,1\right)$ and $\alpha_{2}\in \left[0,1\right)$ are the momentum coefficients for the two statistics and *t* is the iteration round. The statistics are modified as

(10) $$\begin{array}{c} {\tilde{m}}_{t+1}\leftarrow \frac{m_{t+1}}{1-\alpha_{1}^{t+1}}, \end{array}$$

(11) $$\begin{array}{c} {\tilde{v}}_{t+1}\leftarrow \frac{v_{t+1}}{1-\alpha_{2}^{t+1}}. \end{array}$$

As the terms with exponents of t+1 decrease with each time step, the denominators approach one, causing the modification to have a diminishing effect. Finally, we update the parameters as

(12) $$\begin{array}{c} \theta_{t+1}\leftarrow \theta_{t}-\alpha \cdot \frac{{\tilde{m}}_{t+1}}{\sqrt{{\tilde{v}}_{t+1}}+\epsilon }. \end{array}$$

#### 3.3.5. Hyperparameters

In our framework, the learning algorithm and its associated parameters, such as the learning rate, are treated as hyperparameters. The process of selecting optimal values for these parameters is known as hyperparameter search. For this work, we set the ADAM optimizer’s parameters as follows: learning rate $\alpha =1\times 10^{-3}$, $\alpha_{1}=0.9$, $\alpha_{2}=0.999$, and $\epsilon =1\times 10^{-7}$. A detailed exploration of hyperparameter search is beyond the scope of this paper.

### 3.4. NeuroDetect Algorithm

Our proposed NeuroDetect algorithm, to detect input data symbols with low-resolution quantization observations, is summarized in Algorithm 1. The algorithm optimizes the model parameters θ using the input features and one-hot encoded labels during the TP through an iterative process that minimizes the loss in (4). During the DP, the optimized parameters $\theta^{★}$ are utilized to accurately detect signals, ensuring reliable performance.
**Algorithm 1** Proposed NeuroDetect Algorithm for Signal Detection**Require:** *I* training samples ${\left\{\left(Q{\left(Y\right)}^{\left[i\right]},\gamma^{\left[i\right]}\right)\right\}}_{i=1}^{I}$, number of iterations *T*.**Ensure:** $\theta^{★}=\underset{\theta }{\mathrm{argmin}}\;L\left[\theta \right]$ in (4).   1:Initialize model parameters $\theta_{1}={\left\{\beta_{l},\Omega_{l}\right\}}_{l=0}^{4}$.   2:Generate one-hot encoded labels from each $\gamma^{\left[i\right]}$ by using (7).   3:**for** t=1 to *T* **do**   4:    **for** i=1 to *I* **do**   5:        $\lambda^{\left[i\right]}\leftarrow {\mathrm{softmax}}_{\gamma^{\left[i\right]}}\left(f\left(Q{\left(Y\right)}^{\left[i\right]},\theta_{t}\right)\right)$   6:    **end for**   7:    Compute $L[\theta_{t}]$ using ${\left\{\lambda^{\left[i\right]}\right\}}_{i=0}^{I}$.   8:    Back propagate $L\left(\theta_{t}\right)$ and update parameters using (12)   9:**end for**   10:$\theta^{★}\leftarrow \theta_{T}$   11:**Return** $\theta^{★}$

**Remark—Digital Implementation and Bit-Depth Considerations:** NeuroDetect integrates seamlessly with the conventional baseband digital signal processing (DSP) pipelines, requiring no architectural modifications beyond replacing the signal detection module with the NeuroDetect detector. In the proposed receiver architecture, analog-to-digital conversion is performed solely at the RF front end, immediately after the antenna and analog filtering stage, and this is the only point in the receiver chain where quantization occurs. NeuroDetect operates entirely in the digital domain on quantized samples produced by this front-end ADC, and can be efficiently implemented on standard digital baseband processors using conventional fixed-point arithmetic. This design preserves the energy efficiency benefits of coarse RF quantization while maintaining compatibility with the existing DSP infrastructure.

**Remark—Streaming Mode Operation:** NeuroDetect operates as a feed-forward neural network that processes each quantized symbol independently for data detection. It does not require sequence-level buffering or batch processing, making it well suited for streaming (online) operation. A decision is produced with a single forward pass through the network, enabling low-latency symbol detection. The network has a lightweight architecture, enabling efficient implementation in digital baseband hardware.

## 4. Numerical Results

In this section, we present the Symbol Error Probability (SEP) results obtained using our DL-based signal detector, NeuroDetect, for *M*-PSK modulation with *n*-bit quantization. The channel follows a unit-power, circularly symmetric fading model with a Nakagami-*m* distributed magnitude, while the additive noise is complex Gaussian with zero mean and unit variance.

### 4.1. Performance Comparison with the ML Detector

**Figure 4:**
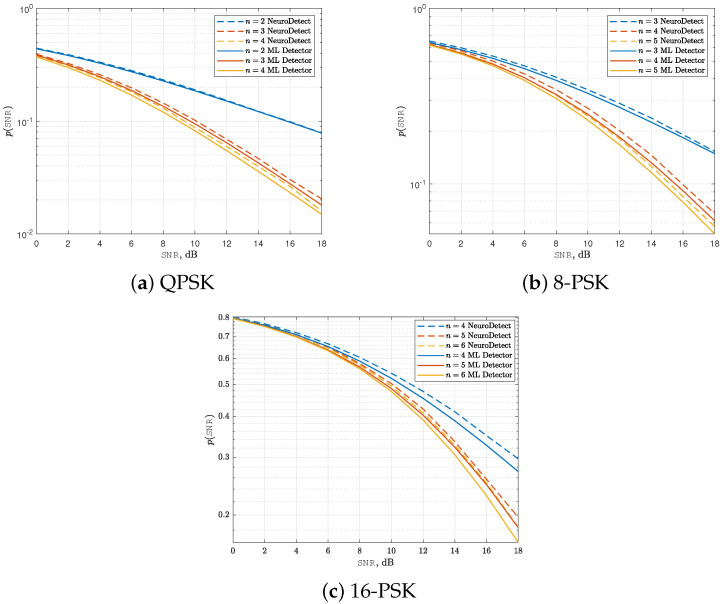
Comparison of the SEP performances of NeuroDetect (no channel knowledge) and the optimum ML detector (full channel knowledge). The number of quantization bits was set to $n=\log_{2}M,\;\log_{2}M+1$, and $\log_{2}M+2$ for M=4,8, and 16.

Figure 4 compares the SEP performance of NeuroDetect with the ML detector from [12] for QPSK, 8-PSK, and 16-PSK modulation schemes. The comparison is made for phase quantization levels of $n=\log_{2}M$, $n=\log_{2}M+1$, and $n=\log_{2}M+2$. The ML detector in [12] is the optimum detection rule if the receiver has access to full channel knowledge. Hence, it serves as the universal lower bound on the SEP performance of NeuroDetect.

As shown in Figure 4, the proposed DL-based signal detector, NeuroDetect, achieves near-optimum performance compared to the ML detector. The main advantage of our NeuroDetect architecture is that this high accuracy is attained without any explicit channel knowledge, whereas the ML detector assumes perfect CSI. The performance gap remains below 12% across all modulation schemes and quantization levels we studied. This result is particularly significant, as accurate channel estimation poses a major challenge in low-resolution quantized receivers. NeuroDetect effectively mitigates this limitation, providing a robust solution for wireless communication systems with low-resolution quantization.

**Remark—Training Overhead:** To quantify the algorithmic overhead introduced by NeuroDetect, we evaluated the amount of labeled data required within each channel coherence interval. Empirically, fewer than 150 labeled symbols are sufficient to reach the near-optimum SEP reported in Figure 4. Assuming a representative urban micro-cell channel with a coherence bandwidth of $B_{c}=1\;\mathrm{MHz}$ and a coherence time of $T_{c}=5\;\mathrm{ms}$ [47], the resulting coherence block contains approximately

$$B_{c}T_{c}\;=\;1\times 10^{6}\;\mathrm{Hz}\;\times \;5\times 10^{-3}\;s\;=\;5\times 10^{3}\;\mathrm{symbols}.$$

Hence, the training overhead of NeuroDetect amounts to

$$\frac{150}{5000}\;\approx \;3\%$$

of the available symbols per block. In other words, 97% of each coherence block remains available for payload data, indicating that the proposed model-free detector can be trained online with negligible rate loss while still achieving near-optimum performance, close to the ML benchmark. This modest overhead, combined with the detector’s feed-forward streaming operation (see the remark on streaming mode operation above), indicates the potential of deploying NeuroDetect in latency-sensitive and bandwidth-constrained wireless systems.

**Figure 5:**
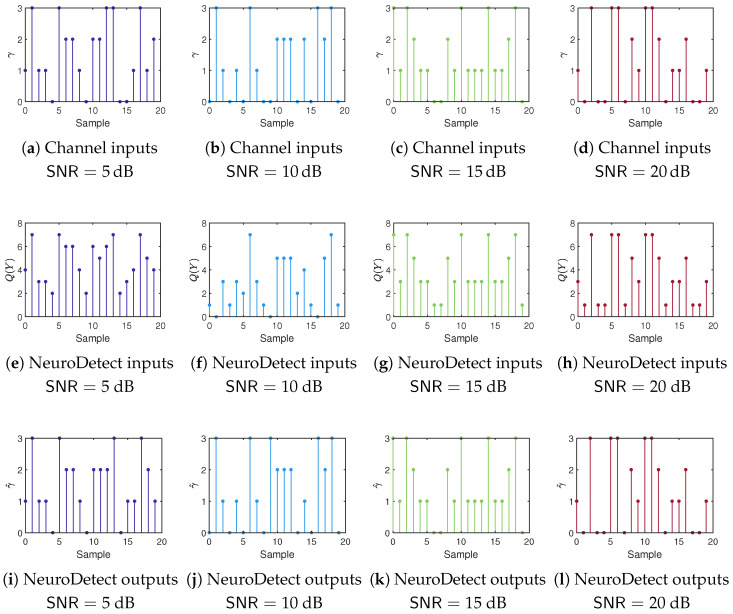
Graphical illustration of time-domain signals under QPSK modulation and 3-bit phase quantization, assuming a fixed channel $h=e^{j\frac{\pi }{6}}$. The channel inputs are represented by transmitted symbol indices γ. The NeuroDetect inputs (or quantizer outputs) correspond to the quantized phase sectors Q(Y), while the NeuroDetect outputs denote the estimated transmitted symbol indices $\hat{\gamma }$.

To provide further insights into the time-domain behavior of NeuroDetect, we provide a graphical illustration of transmitted and received signals in discrete-time using QPSK modulation with 3-bit phase quantization in Figure 5. For the figure, the channel is fixed as $h=e^{j\frac{\pi }{6}}$. Transmitted symbols are randomly generated with a uniform distribution for each SNR level and are represented by symbol indices γ∈{0,1,2,3} in the first row of the figure. The second row shows the inputs to the NeuroDetect detector, which are the quantizer outputs indicating the sector of the received signal. With 3-bit quantization, these outputs take values in {0,1,…,7}. The third row displays the outputs of the NeuroDetect detector, corresponding to the indices of the estimated transmitted symbols. A detection is considered successful when the estimated index matches the transmitted index. Otherwise, a symbol error occurs, for example, see sample 15 at SNR=5 dB.

### 4.2. Effect of Quantization Bits on System Performance

In this part, we examine the impact of quantization bit levels on the SEP performance of the proposed NeuroDetect detector. Specifically, we focus on QPSK modulation and analyze the effect of quantization levels n=2,3, and 4 under Nakagami-*m* fading conditions, considering shape parameters m=1 and m=2.

A key structural property of data detection in low-resolution quantized systems under Nakagami-*m* fading is that the system diversity order (DVO), defined as $\mathrm{DVO}≜\lim_{\mathrm{SNR}\to \infty }\frac{\log p(\mathrm{SNR})}{\log\mathrm{SNR}}$, follows a distinct behavior based on the quantization level. Specifically, $\mathrm{DVO}=\frac{1}{2}$ when $n=\log_{2}M$, and DVO=m when $n>\log_{2}M$. This result, established in [12], characterizes the optimum asymptotic decay rate of the SEP in the high-SNR regime. We will demonstrate that our proposed NeuroDetect detector is asymptotically optimum, as it attains the same diversity order as the ML detector.

**Figure 6:**
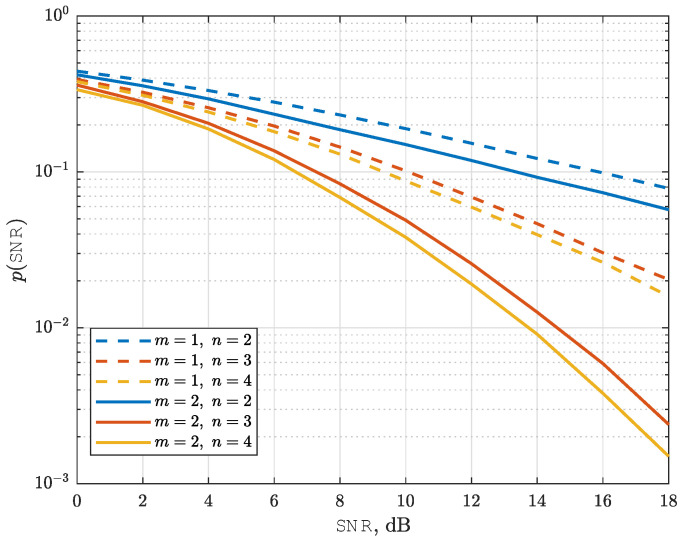
Average SEP curves for NeuroDetect (averaged over random channel realizations) as a function of SNR for QPSK modulation. n=2,3,4 and m=1,2.

Our results are presented in Figure 6. A significant performance improvement is observed for both m=1 and m=2 when the number of quantization bits increases from n=2 to n=3. This behavior aligns with the fundamental structural property of the system’s DVO discussed earlier. Specifically, for n=2, the diversity order remains at $\mathrm{DVO}=\frac{1}{2}$ in both cases for the QPSK modulation, leading to a relatively slow decay of SEP as a function of SNR. However, when *n* increases to 3, the DVO transitions from $\frac{1}{2}$ to *m*, and our proposed NeuroDetect detector achieves the optimum asymptotic SEP decay rate. Further increasing *n* from 3 to 4 yields performance improvements, though with diminishing returns, indicating a saturation effect in the benefits of additional quantization bits.

**Figure 7:**
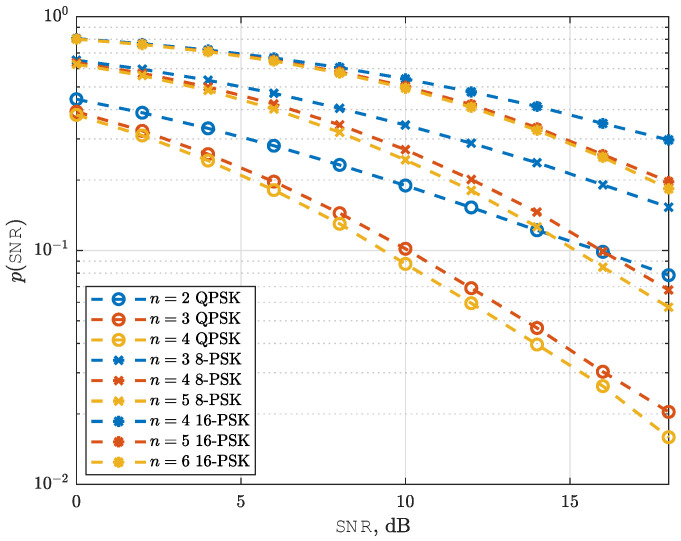
Average SEP curves for NeuroDetect (averaged over random channel realizations) as a function of SNR for different modulation schemes. $n=\log_{2}M,\;\log_{2}M+1,\;\log_{2}M+2$, and m=1.

Figure 7 illustrates the average SEP performance of the NeuroDetect detector, averaged over random channel realizations, as a function of SNR. The results are presented for QPSK, 8-PSK, and 16-PSK modulation schemes, with the Nakagami-*m* shape parameter fixed at m=1, corresponding to the classical Rayleigh fading scenario. The number of quantization bits is set to $n=\log_{2}M$, $\log_{2}M+1$, and $\log_{2}M+2$.

From the Figure 7, it is evident that QPSK with 2-bit quantization, 8-PSK with 3-bit quantization, and 16-PSK with 4-bit quantization exhibit a diversity order DVO of $\frac{1}{2}$. Moreover, increasing the number of quantization bits beyond these levels, specifically QPSK with 3 or more bits, 8-PSK with 4 or more bits, and 16-PSK with 5 or more bits, results in a diversity order of 1, which is equal to *m* in this case. These findings confirm that the NeuroDetect detector is asymptotically optimum, as it attains the same diversity order as the ML detector in the high-SNR regime.

**Figure 8:**
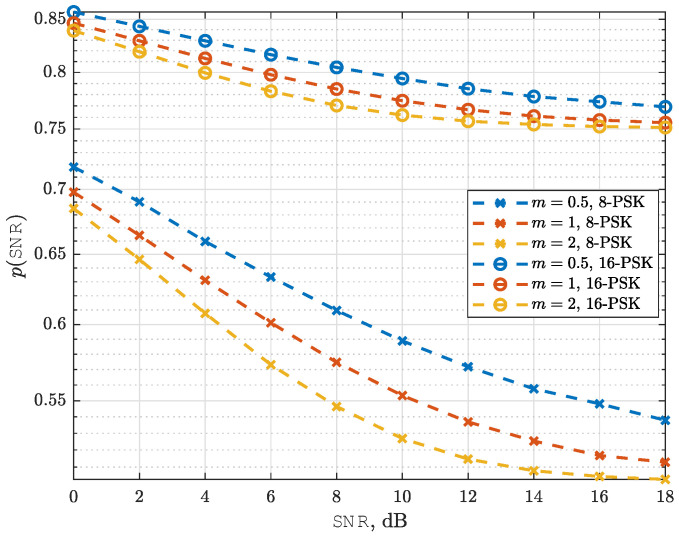
Average SEP curves for NeuroDetect (averaged over random channel realizations) as a function of SNR for 8-PSK and 16-PSK modulation schemes. $n=2<\log_{2}M$, and m=0.5,1, and 2.

In Figure 8, we analyze the average SEP performance of the NeuroDetect detector as a function of SNR for 8-PSK modulation with 2-bit quantization and 16-PSK modulation with 2-bit quantization. The Nakagami-*m* fading parameter is set to m=0.5,1, and 2.

This scenario corresponds to a regime where the number of quantization bits is insufficient to fully resolve the transmitted symbols, leading to an inherent error floor in detection performance. This error floor phenomenon was analytically characterized in [12], which established that the average SEP for 8-PSK with 2-bit quantization is lower-bounded by 0.5, while for 16-PSK with 2-bit quantization, the lower bound is 0.75.

As shown in our results, NeuroDetect exhibits an error floor at high SNR values when $n<\log_{2}M$, aligning with these theoretical limits. Moreover, in both cases, NeuroDetect SEP performance becomes close to these theoretical lower bounds.

### 4.3. Deep Learning Penalty Metrics

We define the increment of the average SEP as a *learning penalty* according to

(13) $$\begin{array}{c} \Psi_{L}\left(\mathrm{SNR},n\right)≜10\log\left(\frac{p\left(\mathrm{SNR},n\right)}{p_{ML}\left(\mathrm{SNR},n\right)}\right), \end{array}$$

where $p\left(\mathrm{SNR},n\right)$ is the average SEP of NeuroDetect with *n* number of quantization bits, and $p_{ML}\left(\mathrm{SNR},n\right)$ is the average SEP of the ML detector with again *n* number of quantization bits.

Similarly, we define the increment of the average SEP for the ML detector as a quantization penalty according to

$$\begin{array}{c} \Psi \left(\mathrm{SNR},n\right)≜10\log\left(\frac{p_{\mathrm{ML}}\left(\mathrm{SNR},n\right)}{p_{\mathrm{ML}}\left(\mathrm{SNR},\infty \right)}\right). \end{array}$$

Using $\Psi_{L}\left(\mathrm{SNR},n\right)$ and $\Psi \left(\mathrm{SNR},n\right)$, the total penalty for the NeuroDetect detector, which is from both the learning penalty and the quantization penalty, is defined according to

(14) $$\begin{array}{ccc} \Psi_{T}\left(\mathrm{SNR},n\right) & ≜ & \Psi_{L}\left(\mathrm{SNR},n\right)+\Psi \left(\mathrm{SNR},n\right)\\ & = & 10\log\left(\frac{p\left(\mathrm{SNR},n\right)}{p_{\mathrm{ML}}\left(\mathrm{SNR},\infty \right)}\right). \end{array}$$

In addition to $\Psi_{T}\left(\mathrm{SNR},n\right)$, we also define *power penalty* as an increase in transmission power to achieve the same average SEP with quantization levels of n−1 and *n* bits, which is given by

(15) $$\Phi_{L}\left(\mathrm{SEP},n\right)=10\log\left(\frac{{\mathrm{SNR}}_{n-1}}{{\mathrm{SNR}}_{n}}\right),$$

where ${\mathrm{SNR}}_{n}$ and ${\mathrm{SNR}}_{n-1}$ are the SNR values required to achieve a target average SEP with *n* and n−1 quantization bits using NeuroDetect, respectively.

**Figure 9:**
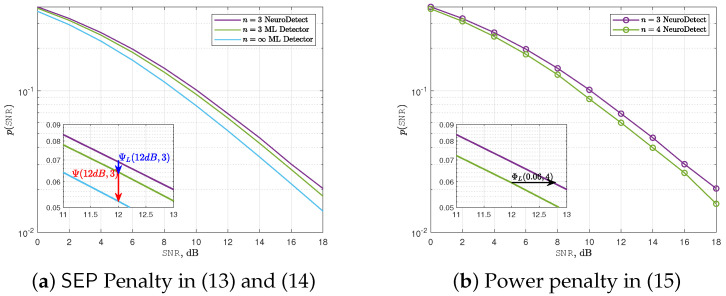
Illustration of deep learning penalty metrics for QPSK.

We illustrate the learning penalty metric for SEP in (13) for NeuroDetect with a 3-bit phase quantizer at an SNR of 12 dB in Figure 9a. Specifically, the SEP learning penalty $\Psi_{L}\left(12\;\mathrm{dB},3\right)$ is 0.31 dB. Due to the quantization process, there is an additional quantization penalty of $\Psi \left(12\;\mathrm{dB},3\right)=0.9$ dB. Therefore, the total penalty when using a 3-bit phase quantizer with NeuroDetect is $\Psi_{T}\left(12\;\mathrm{dB},3\right)=1.21$ dB.

We also illustrate the power penalty metric in (15) for NeuroDetect in Figure 9b. In particular, when the target average SEP is 0.06, we observe a power penalty of $\Phi_{L}\left(0.06,4\right)\approx 0.25\;\mathrm{dB}$ as we change the quantization level from n=3 to 4 for the NeuroDetect. This result implies that to achieve an average SEP of 0.06 with 3-bit quantization, we need 0.25 dB more transmit power than what is required with 4-bit quantization when we use the NeuroDetect.

**Figure 10:**
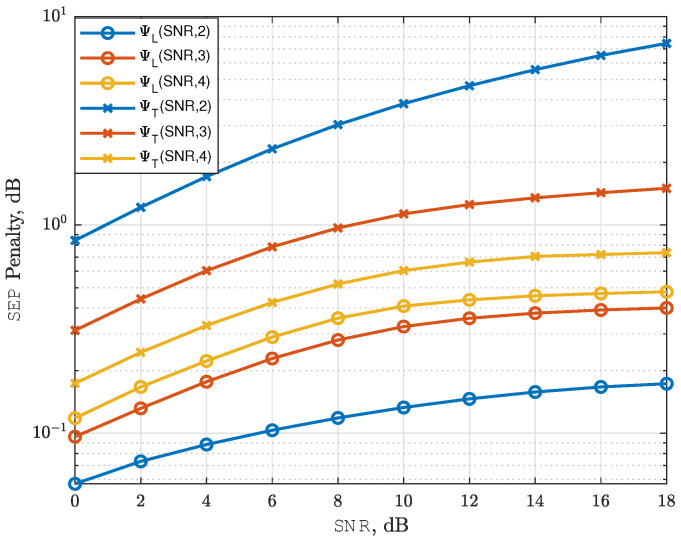
SEP Learning penalty in (13) and SEP learning penalty + quantization penalty in (14) as function of SNR for QPSK.

Figure 10 illustrates the two forms of SEP penalties: the learning penalty from (13) and the total penalty, which combines the learning and quantization penalties, from (14), as a function of SNR for QPSK. When considering the learning penalty, NeuroDetect performs similarly to the ML detector with a learning penalty smaller than 0.4 dB. The gap is smaller and bounded by 0.2 dB when the ADC has $n=\log_{2}M$ bits. This is because, as the number of ADC bits increases, the number of input label categories for NeuroDetect increases to $2^{n}$, while the classification task remains fixed at four classes for QPSK. As a result, mapping $2^{n}$ input labels to four classes becomes more complex, making it harder to learn the relationship between Q(Y) and the most likely transmitted symbol.

We observe a different trend for the total penalty, which also depends on the quantization penalty. As the number of ADC bits increases, the estimation accuracy improves and the quantization penalty decreases. Consequently, the combined learning and quantization penalty decreases with higher ADC bit resolution, eventually converging to the high-resolution case.

### 4.4. Impact of Channel Mismatch Between Training and Data Detection Phases

Finally, we evaluate the robustness of NeuroDetect by employing different channels for training and data detection. Specifically, we introduce phase deviations $\phi =\frac{\pi }{32},\frac{\pi }{16},\frac{\pi }{8},\mathrm{and}\frac{\pi }{4}$ relative to the channel used in the training phase, allowing us to assess the detector’s performance under channel mismatches.

**Figure 11:**
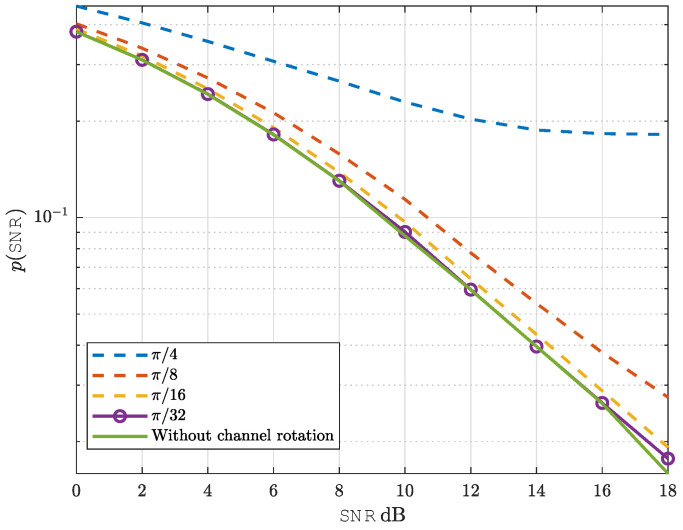
Impact of channel mismatch between training and data detection phases.

Figure 11 shows that NeuroDetect maintains performance comparable to the no-mismatch scenario for channel phase deviations up to $\frac{\pi }{8}$. Within this range, only a constant offset is observed relative to the no-mismatch case. However, for larger deviations, particularly $\phi =\frac{\pi }{4}$, an error floor emerges. In this setting, the mismatch between the channel used for training and the channel encountered during data detection constrains NeuroDetect’s detection performance. In particular, when the channel phase during data detection deviates significantly from the conditions used in training, NeuroDetect encounters *out-of-distribution* samples for which its learned decision boundaries are no longer valid, ultimately resulting in the observed error floor phenomenon.

## 5. Conclusions and Future Work

In this paper, we introduced *NeuroDetect*, a deep learning-based signal detection framework for Nakagami-*m* fading channels with low-resolution quantization at the receiver. By operating in a model-free manner, NeuroDetect eliminates the need for explicit channel knowledge, which is often difficult to estimate under low-resolution constraints. Instead, it leverages training data acquired during each channel coherence interval to learn an optimum signal detection strategy, which is then used for symbol detection during the data transmission phase. Training requires fewer than 150 symbols to achieve near-optimum performance, which corresponds to 3% of a typical coherence block of 5000 symbols (assuming a bandwidth of 1 MHz and a coherence time of 5 ms).

Our numerical findings show that NeuroDetect achieves symbol error performance nearly on par with the maximum likelihood detector, which assumes full channel knowledge. In all examined scenarios, the performance gap remains below 12%. We also show that NeuroDetect attains the optimum asymptotic error decay rates, featuring a fundamental ternary behavior based on the number of quantization bits. These results demonstrate that our proposed model-free learning architecture can closely match the performance of optimum analytical detection strategies, while circumventing the challenge of accurate channel estimation in wireless communications systems with low-resolution quantization.

Our proposed NeuroDetect architecture is already lightweight, comprising five fully connected layers with modest widths determined by the modulation order. An important direction for future work is the development of even more compact architectures that preserve near-optimum detection performance under low-resolution quantization. Such designs would further enhance the practicality of model-free detection in resource-constrained hardware. Investigating this trade-off between model simplicity and detection accuracy is vital to enable scalable deployments of learning-based detectors. Future work may also include hardware-in-the-loop testing to quantify NeuroDetect’s resilience to practical ADC impairments, such as comparator nonlinearities, offset drift, and timing jitter.

## Data Availability

Data are contained within the article.

## Abbreviations

The following abbreviations are used in this manuscript:

ADC	Analog-to-Digital Converter
DAC	Digital-to-Analog Converter
AWGN	Additive White Gaussian Noise
CSI	Channel State Information
DL	Deep Learning
DNN	Deep Neural Network
ML	Maximum Likelihood
MIMO	Multiple-Input Multiple-Output
PSK	Phase Shift Keying
QPSK	Quadrature Phase Shift Keying
SNR	Signal-to-Noise Ratio
LMMSE	Linear Minimum-Mean-Square-Error
BLMMSE	Bussgang LMMSE
MRC	Maximum Ratio Combining
ZF	Zero-Forcing
OSD	One-bit Sphere Decoding
e-MLD	Empirical Maximum Likelihood Detection
MMD	Minimum Mean-Distance
MCD	Minimum Center Detection
FBMCENet	Few-Bit Massive MIMO Channel Estimation Network
FBM-DetNet	Few-Bit Massive MIMO Data Detection Network
OBMNet	One-Bit Massive MIMO Data Detection Network
LoRD-Net	Low Resolution Detection Network
BiLSTM	Bidirectional Long Short-Term Memory
ReLU	Rectified Linear Unit
ADAM	Adaptive Moment Estimation
TP	Training Phase
DP	Data Transmission Phase
SEP	Symbol Error Probability
DVO	Diversity Order
